# Measuring and modeling interventions in aging

**DOI:** 10.1016/j.ceb.2018.07.004

**Published:** 2018-12

**Authors:** Nicholas Stroustrup

**Affiliations:** 1 Centre for Genomic Regulation (CRG), The Barcelona Institute of Science and Technology, Dr. Aiguader 88, Barcelona 08003, Spain; 2 Universitat Pompeu Fabra (UPF), Barcelona, Spain

## Abstract

Many dietary, pharmaceutical, and genetic interventions have been found to increase the lifespan of laboratory animals. Several are now being explored for clinical application. To understand the physiologic action and therapeutic potential of interventions in aging, researchers must build quantitative models.

Do interventions delay the onset of aging? Slow it down? Merely ameliorate some of its symptoms? If interventions slow some aging mechanisms but accelerate others, can we detect or predict the systemic consequences? Statistical and analytic models provide a crucial framework in which to answer these questions and clarify the systems-level effect of molecular interventions in aging.

This review provides a brief survey of approaches to modeling lifespan data and places them in the context of recent experimental work.

## Highlights

• Death involves the final collapse of vital physiological networks, and the timing of this collapse provides a systems-level measure of aging.
• Many of the best statistical models for lifespan data common in the clinical literature are rarely applied in basic research studies.
• Multivariate regression models allow differences between experimental replicates to be explicitly measured and accounted for when estimating the effect of interventions.
• Semi-parametric models allow interventions to be studied with fewer implicit assumptions regarding the empiric data.
• Competing risk models and mixture models provide formal frameworks for reasoning about multi-causal, multi-outcome aging processes.

**Current Opinion in Cell Biology** 2018, **55**:129–138

This review comes from a themed issue on **Differentiation and disease**

Edited by **Katja Röper** and **Xosé R Bustelo**

For a complete overview see the Issue and the Editorial

Available online 10th August 2018

**https://doi.org/10.1016/j.ceb.2018.07.004**

0955-0674/© 2018 The Author. Published by Elsevier Ltd. This is an open access article under the CC BY license (http://creativecommons.org/licenses/by/4.0/).

**Figure 1:**
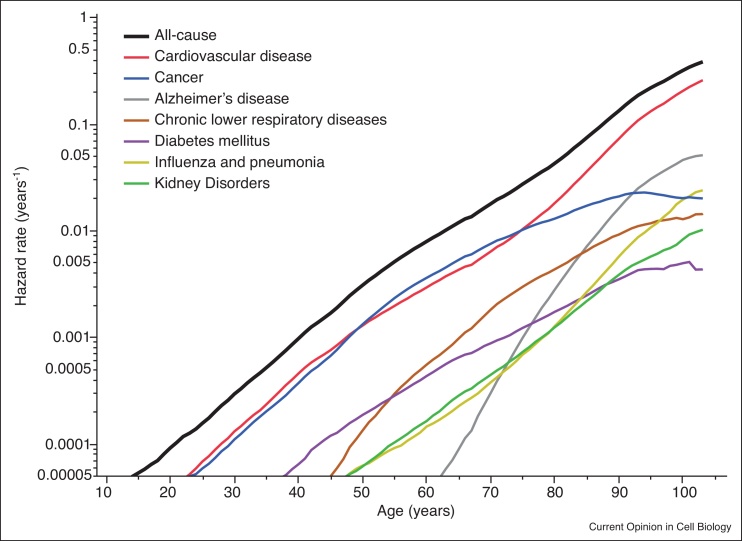
**Systems-level measurement of complex physiological processes**. The risk of death from the seven most frequent causes of non-accidental death is shown, corresponding to 70% of all deaths reported in the USA in 2015 [1]. Cause-specific risks *(colored lines)* sum up to produce the all-causes hazard function *(black)*. Each cause exhibits distinctive age-dependent effects, though substantial correlations exist among the causes.

Douglas Adams once said that ‘There is an art to flying, or rather a knack. The knack lies in learning how to throw yourself at the ground and miss.’ Immortality requires learning a similar knack: you must first be born and then subsequently avoid dying forever. This is challenging in part because there are many different causes of death to avoid — accidents, infections, cancer, heart disease, neurodegenerative disorders — and because our bodies slowly change in ways that make most of these causes of death increasingly probable (Figure 1). As the risks of occurrence for different diseases increase, their contributions add up to produce a doubling in all-cause mortality risk approximately every eight years [1]. The specific physiological changes driving these increases in disease-specific mortality risk remain uncertain, motivating intense research into the molecular, cellular, and systems biology of aging.

The mathematics and statistics used to measure changes in the risk of death are non-trivial and can often appear obscure or arcane to experimental biologists. Yet a basic understanding of the models used to analyze lifespan data is broadly useful. In a clinical setting, quantitative models clarify the challenges ahead for proponents of radical lifespan extension, as delaying or eliminating individual causes of death will statistically yield only modest incremental increases in lifespan. For example, it is estimated that curing all cancers would produce less than four years of extended lifespan [2]. In a basic research setting, lifespan data usually provides the strongest evidence for any molecular mechanisms’ involvement in aging. A working understanding of statistical models allows researchers to critically evaluate this evidence.

## Describing aging using hazard and survival functions

Analysis of lifespan data is grounded in study of two related mathematical functions — the survival curve and the hazard function. The hazard function provides an intuitive measure of the risk of death, describing the probability that a typical individual who is currently alive will soon die. This probability is much higher in older individuals compared to younger ones, an observation usually interpreted as evidence of some physiological weakness or susceptibility to death shared among old individuals not present in the young. Over the last forty years, it has become very clear that this increase in risk does not emerge from some universal natural law. The shape of hazard functions is a product of evolutionary forces and varies enormously between species [3]. In fact, one mammal, the naked mole rat, has recently been shown to exhibit a nearly constant hazard function [4]. Formally, the hazard function is defined as a conditional probability, *h*(*t*) = lim *P*(*T* ≤ *t* + Δ*t*|*T* > *t*) as Δ*t* → 0, and is usually estimated and plotted as the rate *h*(*t*) = *P*(*t* < *T* ≤ *t* + Δ*t*)/*P*(*T* > *t*).

The survival curve is a separate but closely related function that describes the fraction of a population that remains alive over time. At the start of an observational period, this fraction is one and then drops each time an individual dies. The survival function is formally defined as the cumulative probability of remaining alive, *S*(*t*) = *P*(*T* > *t*), and is related to the hazard function by $S(t)=\exp\left(-\int_{0}^{\tau }h(\tau )d\tau \right)$. Though the hazard function often provides a clearer visualization of patterns in mortality, any model of lifespan data can be equivalently stated in terms of the survival function.

## Identifying changes in lifespan with non-parametric methods

The analysis of lifespan data usually involves the application of a non-parametric test used to identify statistically significant changes in lifespan. Common methods include the log-rank, Wilcox, and the modified Kolmogorov–Smirnov (KS) tests, all of which ask whether two population’s lifespan correspond to the same underlying survival and hazard function. These tests make relatively few assumptions about the statistical properties of the underlying lifespan data, and so have remained in continuous use for decades without substantial modification. However, this lack of assumptions limits the types of conclusions that can be drawn — most non-parametric tests can show that lifespan has been altered but not how it has been altered. Recently, non-parametric approaches have been developed to distinguish changes in mean lifespan from changes in the variation in lifespan, as part of a pace-shape framework [5^•^].

## Modeling the hazard function with parametric models

**Figure 2:**
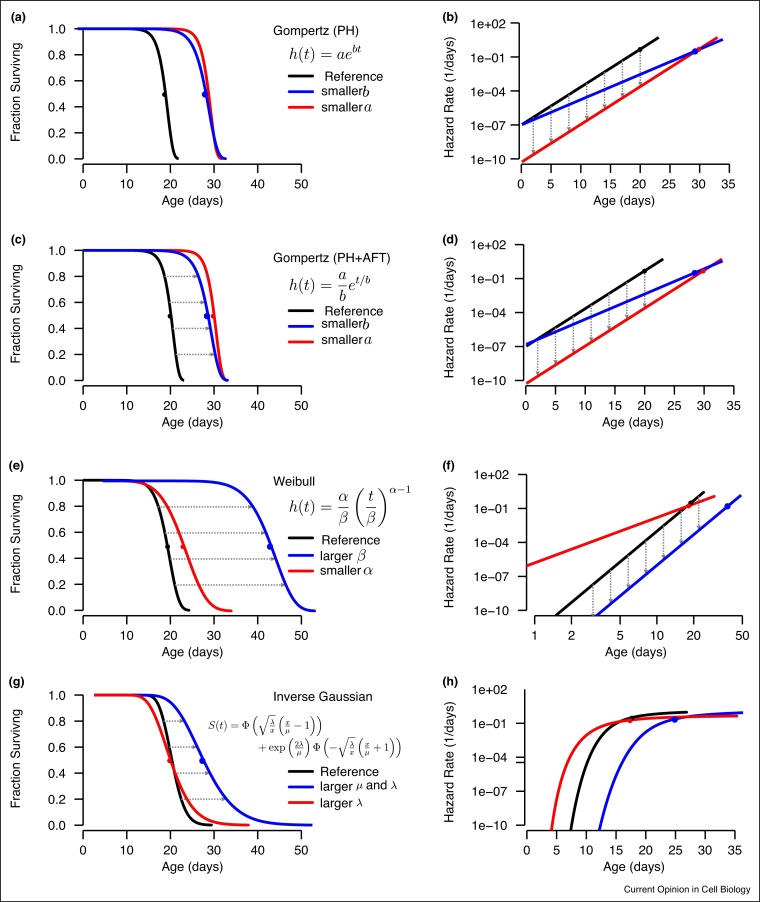
**Parametric models of lifespan data**. Various simple functions have been proposed to approximate empiric lifespan data, shown as models of survival functions *(left)* and *(right)* hazard functions. Median lifespan is marked as a single point on each corresponding curve. Geometric regularities in the influence of various parameters are marked with gray arrows. **(a,b)** The Gompertz distribution is commonly employed with the parameter *a* that determines the risk of death in animals of zero age and *b* that determines the rate of increase in risk over time. **(c,d)** The alternate parameterization of the Gompertz model removes the implicit and often missed time-scale dependence of the *a* parameter, allowing changes in initial mortality and changes in doubling time to be isolated, and allowing the Gompertz function to model both changes in proportional hazards and changes in timescale. **(e,f)** The Weibull distribution models hazard functions that increase as a polynomial of time (in contrast to the exponential increase assumed in by the Gompertz model). Weibull hazard functions therefore form straight lines when plotted on log–log axes, rather than log-linear axes. **(g,h)** Inverse Gaussian distributions exhibit inherently decelerating hazard functions, and provide a link between lifespan data to the theory of Weiner Processes and Brownian motion.

Parametric models go beyond non-parametric models by asserting that lifespan data can be accurately described by simple mathematic functions with a small number of free parameters. Where this assumption holds, parametric functions allow researchers to reason about survival and hazard functions in simple, intuitive geometric terms. For example, the commonly used Gompertz model assumes that populations exhibit hazard functions that increase exponentially over time (Figure 2a–d), following a straight line on a log-linear plot.

Different models have been proposed to explain why physiological aging processes might yield lifespan distributions conforming to simple parametric forms. The more quantitative of these theories draw on a mix of reliability theory [17], complex networks theory [18], and statistical physics [19]. Most common parameterizations include (or can be re-parameterized to include) a single parameter that uniquely governs the time-dependent increase of the hazard functions, called a ‘timescale’ or ‘rate’. Examples include the Gompertz *b* parameter, the Weibull *β* parameter, and the Inverse Gaussian *λ* parameter. Such timescale parameters are generally interpreted as measuring a population's ‘rate of aging’, which is some average speed at which physiologically young individuals change into old individuals. Empiric data can be used to distinguish an intervention's effect on this rate from other parameters. In this way, interventions can be categorized according to their distinct effects on different parameters [20, 21, 9, 22, 23•].

The usefulness of any parametric model depend crucially on whether empiric data do in fact exhibit such patterns, and the Gompertz model has been shown to provide a reasonable approximation for some human populations [6,7] as well as some invertebrate populations [8,9]. Yet, Gompertzian patterns should not be assumed *a priori*, as the shape of hazard functions varies enormously between species [3] and despite its popularity the Gompertz model is frequently out-performed by several other two-parameter distributions. These include the Weibull model [10] that assumes a polynomial increase in the risk of death over time (Figure 2e,f), as well as the inverse-Gaussian model (Figure 2g,h) which notably has a compelling theoretic grounding in the statistical physics of random walks [11,12]. Other parametric alternatives include Gompertz–Makeham, log-logistic, and log-normal models. Parameter estimates can be obtained by a variety of methods [13,14], with maximum-likelihood estimation approaches [15] almost always producing the most accurate results [16].

The major limitation to parametric methods is that for most data sets, there does not exist a single unambiguously best parametric form. In cases where different parameterizations can equally well approximate a data set, the different parameterizations will provide multiple, discordant interpretations. Several reasons for this empiric ambiguity are described in subsequent ‘frailty’ and ‘competing risk’ sections.

## Modeling changes in lifespan with semi-parametric methods

Semi-parametric models improve on parametric approaches by eliminating the need for risky assumptions about the shape of survival and hazard functions. Semi-parametric models parameterize only the difference between two survival curves — the action of the intervention itself. In this way, semi-parametric models provide a more flexible means for evaluating interventions in aging, often with practically equivalent statistical power as parametric methods [24^•^].

**Figure 3:**
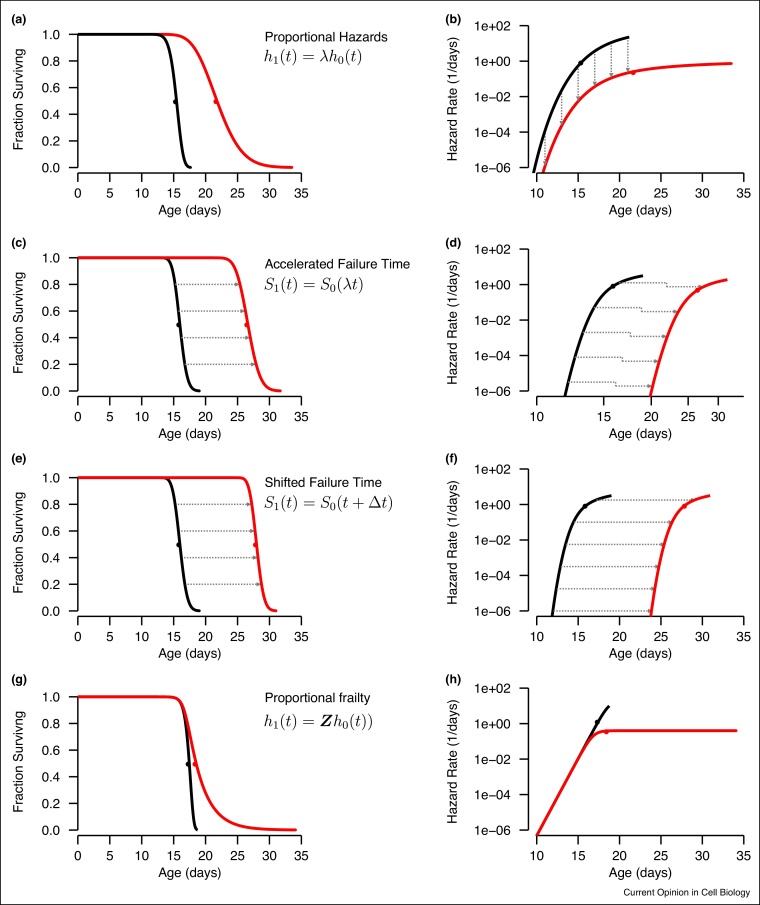
**Semi-parametric models of lifespan data.** Semi-parametric models describe differences between populations in a way that does not depend on any particular parametric form of the survival curve of hazard function. **(a,b)** Proportional hazards functions assume that two populations’ hazard functions are offset by a constant ratio. **(c,d)** Accelerated Failure time models assume that two populations’ survival curves are related by a temporal scaling, corresponding to simultaneous shift of the hazard functions, up and to the right such that *h*_1_(*t*) = *λh*_0_(*λt*). **(e,f)** Accelerated Failure time models are easily modified to model populations whose survival distributions are shifted (rather than scaled) in respect to time. **(g,h)** The existence of heterogeneity within a population in respect to the risk of death produces a deceleration, or leveling-off of the hazard function and a corresponding long-tail of the survival function.

The two most common families of semi-parametric models are Proportional Hazards (PH) models and Accelerated Failure Time (AFT) models. Proportional hazards models assume interventions alter the hazard function to produce a time-independent proportional change in the risk of death. In practice, this means that PH models require that an intervention produces a vertical shift of the hazard function when plotted on log-linear axes, equivalent to a change in a Gompertz *a* or a Weibull *β* parameter (Figure 3a,b). PH models are formally defined by the relation *h*_1_(*t*) = *λh*_0_(*t*), where *h*_1_(*t*) is the hazard function of a population exposed to some intervention with *h*_0_(*t*) as the control group. Accelerated failure time models, in contrast to PH models, assume that interventions produce a temporal rescaling of aging, stretching or compressing survival curves such that *S*_1_(*t*) = *S*_0_(*λt*) (Figure 3c,d). This scaling is equivalent to a change in the Gompertz *b* or Weibull *β* parameters. AFT models offer an intuitive physical interpretation, that interventions extend lifespan by decreasing the rate of the underlying molecular or cell biologic processes that determine the timing of death.

Semi-parametric models are widely used in clinical research and therefore a deep literature exists exploring their behavior in diverse contexts [25,26]. Multivariable methods such as Cox regression and Buckley–James regression allow multiple influences on lifespan to be considered, for example where environmental factors differ between experimental replicates of a single intervention [27^•^] or when multiple interventions are applied simultaneously. PH and AFT models often provide very good approximations of empiric data in a variety of organisms, including yeast [28^•^], flies [5^•^], nematodes [23^•^,29^•^], and mice [30,31]. A variety of approaches exist to identify and compensate for situations where assumptions are not met, including segmenting time or allowing continuously time-varying hazard ratios [32]. Additive hazards (AH) models, assuming *h*_1_(*t*) = Δ*h* + *h*_0_(*t*) have also been suggested [33].

AFT models, unlike PH models, produce residual distributions that take the same time units as the lifespan data provided. This allows residuals to subsequently be used as a time-standardized lifespan distribution for qualitative comparisons between different populations [29^•^] and a convenient means for handling confounding effects of environmental factors [34]. In cases where interventions do not slow the aging process, but rather delay it by a fixed interval to produce a rigid shift of the survival curve, interventions can be modeled using similar methods assuming *S*_1_(*t*) = *S*_0_(*t* − Δ_*τ*_) (Figure 3e,f).

## Accounting for heterogeneity within groups with frailty models

Basic parametric forms like Gompertz and Weibull are often employed with the implicit assumption that all individuals in a population age according to the same parameters. This assumption is rarely justified by experimental data. Even within isogenic populations of laboratory animals housed in controlled laboratory conditions, subpopulations are observed to age in distinctive ways [35, 36, 37, 38, 39, 40••]. Heterogeneity can also be produced when individuals respond unevenly to an intervention [41, 42•, 43].

The effect of the heterogeneity on the hazard and survival functions can be modeled even when it cannot be explicitly measured. This is accomplished by assuming the effect of the unmeasured heterogeneity takes a simple parametric form as it varies between individuals. This heterogeneity is then incorporated as an extra parameter in parametric or semi-parametric models and referred to as a ‘frailty’ or ‘random effect’ term. A two-parameter Gompertz model, for example, can be modified to account for heterogeneity in respect to its *a* parameter by adding a single additional parameter, *σ*, to represent the variance of an gamma-distributed *Z* random variable, such that the hazard function becomes *h*(*t*) = *Z*(*σ*)*a*/b exp(*t*/*b*) [44].

Heterogeneity of this kind produces a stereotypic, geometric effect on hazard functions — a progressive deceleration of hazard functions relative to the basic underlying parametric form [45]. This deceleration can be large enough to produce a plateauing (flattening) of hazard functions (Figure 3g,h). This deceleration arises as a consequence of high-frailty, high-risk subpopulations dying earlier than low-frailty and low-risk sub-populations. As the high-risk individuals die off, the remaining population increasingly consists of relatively low-risk individuals. This change in the populations’ composition counteracts the increasing risk of each individual, producing a quasi-stationary state in which the hazard rate appears flat [46]. Decelerating hazard functions are observed in most model-organism lifespan data. In some data sets the effect is subtle, but in many cases late-life deceleration is a dominant feature that limits the practical application of simple two parameter Gompertz or Weibull models [45, 47, 48, 29•]. Frailty-associated heterogeneity also confounds efforts to experimentally identify a ‘true’ distributional form of lifespan distributions produced by aging. Simple parametric models are most easily distinguished by their behavior at late ages, at the tail of the parametric probability distribution. Heterogeneity masks the underlying form of these tails, undermining biological interpretations that depend on empiric justification of specific parametric forms [29•, 49].

## Accounting for multiple causes of death with competing risk models

**Figure 4:**
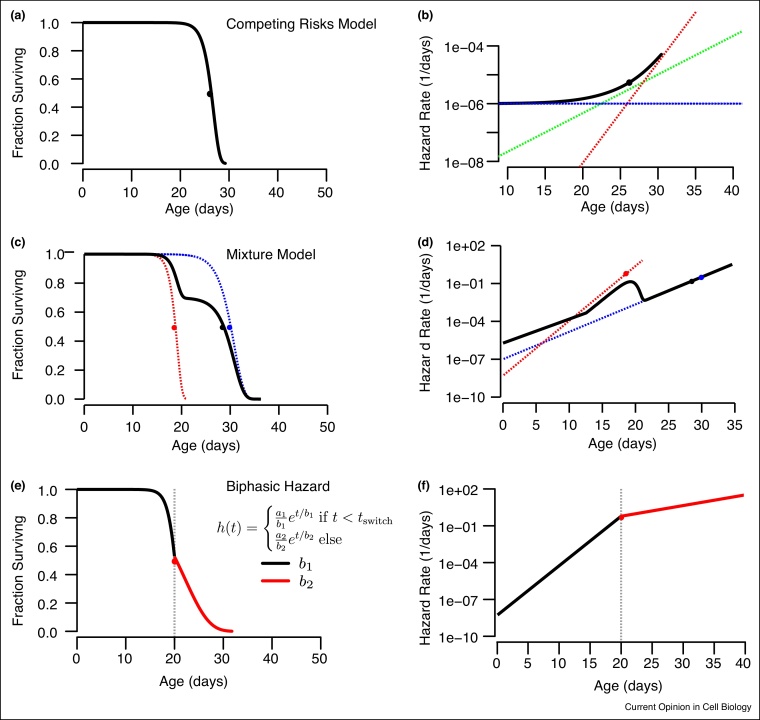
**Modeling different types of heterogeneity.** Several techniques exist to describe heterogeneity between individuals within a population and over time during the aging process. **(a,b)** Competing risk models describe the effect that multiple causes of death have on a population's hazard function. Here, three statistically independent causes of death exhibit different temporal dynamics. One cause *(blue)* shows a constant risk over time, another cause *(green)* increases slowly with age and a third *(red)* increases rapidly later in life. The cause-specific hazard functions of each cause sum to produce the all-cause hazard function and survival curve *(black)*. **(c,d)** In come cases, the cause of death of an individual may be predetermined early in life, producing distinct subpopulations dying from distinct causes of death. Here, thirty percent of individuals die early according to one cause of death *(red)* and the remainder die according to a second cause *(blue)*. In this case, the hazard functions do not strictly add. **(e,f)** Survival and hazard functions can be modeled with a ‘change-point’ that separates distinct phases of aging. Though geometrically compelling, the biological interpretation of segmented hazards is often problematic, as the break-point time must be uncorrelated with each individual's death time.

Competing risks (CR) models explore the idea that though an organism can die only once, it remains at risk of dying from multiple possible causes up until the moment that one particular cause kills it. These models are most intuitively applied for individuals obviously suffering from several potentially fatal diseases — for example cancer patients with cardiovascular conditions [50]. CR models explore the way different causes of death interact to determine survival and hazard functions, and provide a framework in which interpret situations where interventions do not effect all causes of death equally [51]. Competing risk models involve data where each death time *T*_*i*_ is paired with a label *C*_*i*_ describing the cause of death. This allows an intuitive decomposition of the all-cause hazard into the sum of several cause-specific hazard functions, *h*(*t*) = ∑_*i*_*h*_*i*_(*t*) = ∑_*i*_ lim*P*(*t* ≤ *T* + Δ*t*|*T* > *t*, *C* = *C*_*i*_) as Δ*t* → 0 [26], shown in Figures 1 and 4 a,b.

Some CR models assume that all individuals share the same risks for each cause of death, with physiological changes shared among all individuals nevertheless producing qualitatively distinct outcomes (Figure 4a,b). Other CR models, often called ‘Mixture models’, assume instead that sub-populations differ in their risks for different causes, with the most extreme case being when causes of death are predetermined early in life (Figure 4c,d). The empiric validity of these assumptions is of particular contemporary interest given the many types of distinct subpopulations now being identified within isogenic populations [52••, 53, 54, 55].

The assumptions required to formulate CR models are now used as a rigorous starting point for experimental researchers attempting to understand the physiological basis of aging. For example, the temporal scaling of lifespan distributions has been interpreted using a CR framework as suggesting that many interventions act equivalently on all physiologic determinants of the risk of death [29^•^]. More generally, competing risk models provide a quantitative framework for exploring the relationship between different aging mechanisms and their integrative effect on lifespan. The identification of distinct causes of death in model systems [52^••^,56^•^,^57^] opens the door to exploring the mechanistic basis of competing risk phenomena.

## Describing multi-stage aging processes

The physiologic processes that transform young individuals into middle age individuals may be distinct from the physiologic processes transform middle age individuals into elderly individuals. Several lines of research are exploring such distinctions based on a variety of observable differences the aging processes working in young and old individuals, including transitions in gut permeability [58], changes in rate of morphologic properties like body size and texture [40^••^], transcriptomic changes [59, 60, 61•], protein aggregation [62,63], and differences in susceptibility to early-life [52^••^] or late-life [64] bacterial infection. In some cases, the effect these phases have on lifespan may be minor, and specialized statistical techniques may not be needed. However, when individuals in different states exhibit distinctive risks of death, multi-state models often called ‘illness-death’ models will be applicable [65].

## Change-point models and segmented hazard functions

A class of models have been proposed that separate aging into distinct phases relative to a landmark, or ‘change-point’, specified in chronological time [66, 67, 68, 69]. (Figure 4e,f). The biological interpretation of these models is often problematic. For a sharp transition to be observed in a population's hazard function, all individuals alive at the change-point must simultaneously switch to the new phase. The simplest mechanism this would be an external factor such as a shift in environment or diet, effecting all individual at the change-point. The segmented hazard function then would reflect an aspect of the environment and not aging itself. Absent such external factors, the change-point would need to be determined by some intrinsic physiologic process. This process would need to be independent of the processes determining lifespan, as individuals variable in their lifespan would nevertheless need to switch synchronously between phases. A more physiologically plausible explanation would be that segmented hazard functions arise not from a multi-phasic aging process but instead from the distinct contributions of unidentified subpopulations to the population hazard function, as described by competing risks and mixture models.

Biphasic hazard models in other cases may simply represent an over-fitting or mis-fitting of empiric data. For example, an apparently biphasic Gompertzian hazard function may be better explained by a single-phase inverse Gaussian distribution or a single-phase Gompertzian distribution with an extra parameter correcting for the effects of frailty.

## Summary

Aging research is undergoing a period of rapid discovery and characterization of genetic, pharmaceutical, and dietary interventions in aging. Several of these therapies are being explored for translational potential, and lifespan data from human clinical trials may soon be available in which patients’ survival is altered by molecular perturbation of basic aging processes. This growing abundance of lifespan data demands the thoughtful application of statistical methods.

For these projects, familiar analytic techniques should be re-evaluated. In particular, researchers should recognize that the Gompertz hazard parameterization became standard decades ago, long before the high-resolution data needed to validate it became available. Experimentalists should, as an alternative, consider frailty-corrected Gompertz distributions or semi-parametric methods like AFT or PH regression. Finally, competing risks and mixture models should seriously considered in situations where multiple aging processes may influence one or more outcomes in aging. Employing a diverse set of analytic approaches, experimentalists can move beyond humble significance testing to instead use lifespan data as a versatile means for studying the physiological dynamics of aging.

## References and recommended reading

Papers of particular interest, published within the period of review, have been highlighted as:

• of special interest
•• of outstanding interest
